# Comparison of the early development of the professional values for nursing students in the traditional program and the second‐degree program: a longitudinal study

**DOI:** 10.1002/nop2.1150

**Published:** 2021-12-16

**Authors:** Hui‐Ping Lin, Tsai‐Hsiu Chang, Hsiu‐Fang Lee, Yu‐Hsia Tsai, C.W Chang, Hui‐Chu Chiang, Hsueh‐Erh Liu

**Affiliations:** ^1^ Department of Nursing Taoyuan Branch of the Ministry of Health and Welfare Taoyuan Taiwan; ^2^ Graduate Institute of Clinical Medical Sciences College of Medicine Chang Gung University Taoyuan Taiwan; ^3^ Department of nursing Hungkuang University Taichung Taiwan; ^4^ Department of Nursing Chang Gung Memorial Hospital Tao‐Yuan Taiwan; ^5^ Department of Nursing College of Nursing Chang Gung University of Science and Technology Taoyuan Taiwan; ^6^ School of Nursing College of Medicine Chang Gung University Tao‐Yuan Taiwan; ^7^ Department of Cardiovascular Medicine Chang Gung Memorial Hospital Linkou Taiwan; ^8^ Assistant Research Fellow at Division of Pediatric Endocrinology & Genetics Department of Pediatrics Chang‐Gung Memorial Hospital TaoYuan Taiwan; ^9^ School of Nursing National Taipei University of Nursing and Health Sciences Science Taipei Taiwan; ^10^ Department of Rheumatology Chang Gung Memorial Hospital Taoyuan Taiwan

**Keywords:** longitudinal study, nursing students, professional value

## Abstract

**Aim:**

This study aims to compare the early development of professional value between the students in the traditional programme (BSN) and those in the accelerated BSN (ABSN) programmes.

**Design:**

A longitudinal design was conducted.

**Methods:**

Data were collected from three schools of nursing during one academic year. A total of 117 BSN students and 101 ABSN students completed the survey of demographic information and the Nurses’ Professional Values Scale–Revised questionnaires. All data were analysed by IBM SPSS‐Statistics 22.

**Results:**

Results showed that, in the beginning of the first professional nursing course, both students in the BSN and the ABSN programmes reported similar level of professional values. However, after one academic year, the changes in the professional value varied both between these two programmes and among the three different nursing schools. The increased professional value in school A represented the possibility for students to improve during their first‐year professional nursing programme. As educators, we should redesign our teaching strategies according to the different conditions of students in each programme.

## 1 INTRODUCTION

Nurses are the largest group in the healthcare system. Although lots of strategies have been implied, nursing shortage is still a worldwide challenge in the healthcare system, especially during the pandemic of the COVID‐19 recently (Spurlock, 2020). In order to increase the manpower, four nursing schools started the accelerated BSN (ABSN) programmes at 2016 by the assignment from the Ministry of Education, Taiwan, ROC. One more school joined this programme at 2018.

The students in the ABSN programmes are expected to reach similar nursing professionalism outcomes at graduation as students those in the BSN programme, although various differences are known to exist, such as stress (Kowitlawakul et al., 2013) and curriculum plan (Payne & Mullen, 2014).

The baccalaureate education in nursing includes the development of the professional values and value‐based behaviours. The values can be taught, modified and promoted directly or indirectly through the education (Leners et al., 2006). Will the students in the BSN and the ABSN programmes develop similar professional value, which has been identified as the core of the professional nursing behaviours?

## 2 BACKGROUND

Most of the studies explored the professional values of student nurses used cross‐sectional study with comparisons in different academic year (Shafakhah et al., 2018), across the academic years (Caldwell & Katie, 2016), types of educational programme (Fisher, 2014; Martin et al., 2003), geographic location (Bang et al., 2011) and cultures (Alfred et al., 2013; Alkaya et al., 2018). However, inconsistent results were found. For example, when compared with the status at entry, a significant increase in professional value was found at present (Kantek et al., 2017; Lin et al., 2010), at graduation (Fisher, 2014; Leners et al., 2006) or after completed an ethics course (Knecht et al., 2019). Nevertheless, some studies demonstrated no significant difference of the professional value between students’ entry and exit (Posluszny & Hawley, 2017) or at different school years (Caldwell & Katie, 2016).

In regard to the types of programmes, some studies found that the BSN students had higher professional values than the students with the associate degree in nursing (Fisher, 2014; Martin et al., 2003). Conversely, some studies showed that the BSN students reported less professional values than the students in the diploma and the ABSN programmes (Feller et al., 2019). There were also studies demonstrated no statistical difference was observed in the overall nursing values between the traditional and the accelerated graduating senior BSN students (Astorino, 2006), or among nursing students, new graduates and seasoned nursing profession (LeDuc & Kotzer, 2009). One study observed that the student in the ABSN reported a higher professional value than the students with associated degree (Feller et al., 2019). Nevertheless, nurses reported a lowest professional value when compared with the student nurses (Poorchangizi et al., 2019b), clinical nurses and nursing instructors (Bijani et al., 2019).

Professional value in nursing students was significantly different between six regions throughout the Iran (Poorchangizi et al., 2019b). In regard to the issue of culture, students from US and Taiwan reported a similar high levels of the overall professional value, but they weighed differently on certain individual items (Alfred et al., 2013). All these diverse results indicate the necessity of further exploration, especially among different programmes.

Students of the ABSN in Taiwan firmly believed that the choice of nursing profession was the right direction for a brighter future and they looked positively at the problems they faced and sought solutions during their first year in the ABSN programme (Yang et al., 2019). Will these characters affect their process of professionalism? Therefore, we included the first‐year students in the ABSN programme and the second‐year students in the BSN programme, who just started their initial learning phase of nursing profession as the samples for the present study. The purpose of this study was to compare the early development of professional value between the ABSN programme and the BSN programme within three schools during one academic year.

## 3 METHODS

### 3.1 Research design and setting

This is a prospective, multi‐site, longitudinal study. Data were collected from three nursing schools from August 2017 to July 2018. Participants were contacted twice for data collection during an academic year. Three of the five schools providing both the ABSN programme and BSN programme agreed the research groups to contact with their students in each programme.

### 3.2 Samples

The inclusion criteria of samples were the first‐year ABSN students or the second year BSN students since they took the same first professional nursing course in this specific academic year, and those who agreed to receive data collection twice during study period. Before the research started, we obtained the permission from the IRB (201701656B0) and from each school first. Then, we contacted with the class leaders in each programme and arranged meeting schedules with the whole classes. We explained the purpose of the study and issues of voluntary participation. Once subjects agreed and signed the informed consents during that meeting, questionnaires were distributed to them immediately. Researcher waited and collected questionnaires after the students completed. A total of 101 students in the ABSN programme and 117 students in the BSN programme completed and returned their questionnaires during the study period. A power of 0.89 was achieved by this data set.

### 3.3 Instruments

#### 3.3.1 Demographic data of subjects

The collected demographic data included age, gender, religion, marriage status, types of educational programmes and pre‐ABSN educational programme.

#### 3.3.2 Nurses’ Professional Values Scale–Revised

Based on the American Nurses Association's (ANA) code of ethics with interpretive statement, Weis and Schank (2009) revised the Nurses Professional Values Scale (NPVS) into the Nurses’ Professional Values Scale–Revised (NPVS‐R) in order to update the measurement of the professional values (Weis & Schank, 2009). Expert validity and *Cronbach's alpha* (0.70–0.92) were conducted, and the validity and reliability were verified upon this updated scale. It consists of 26 items that address the following subscales as caring; professionalism; activism; trust; and justice.

The responses are based on the 5‐point Likert scale, with the score of each item ranging from 1 (“not very important”) to 5 (“very important”). The range of total score is between 26 and 130, a higher score indicates the respondent's greater familiarity with the professional values. NPVS‐R has been translated into many languages in order to measure the professional nursing values in different cultures. In the present study, the range of *Cronbach's alpha* was 0.73–0.84.

### 3.4 Statistical analysis

All data were handled by the IBM SPSS‐Statistics 22. The descriptive statistics were conducted to describe the characteristics of each variable. *Paired t* test, *t* test and ANOVA were performed to verify the expected relationships among variables. A *p* value <.05 was set as significant.

## 4 RESULTS

### 4.1 Characteristics of the samples

Subjects in the BSN programme within three sites were characterized as female, no specific religion, unmarried and unemployed (Table 1). Subjects in the ABSN programme have similar characteristics, except age. When all subjects were grouped as the BSN and the ABSN, *t* test showed that subjects in the ABSN programme were significantly older than subjects in the BSN programme (27.57 vs. 20.83, *t* = 17.53, *p *< .001). Nevertheless, comparison of age for subjects in the BSN programme within three schools showed no differences among them (*F* = 2.017, *p *> .05). Similar results were also found for the subjects in the ABSN programme (*F *= 0.29, *p *> .05).

**TABLE 1:** Characteristics of samples

Variables	A				B				C			
	BSN	%	ABSN	%	BSN	%	ABSN	%	BSN	%	ABSN	%
Gender												
Male	12	34.3	1	3.8	2	5.9	9	21.4	9	18.0	8	24.2
Female	23	65.7	25	96.2	31	91.2	33	78.6	41	82.0	25	75.8
Religion												
None	18	51.4	18	69.2	27	79.4	29	69.0	34	68.0	21	63.6
Buddhism	7	20.0	2	7.7	4	11.8	3	7.1	4	8.0	3	9.1
Taoism	2	5.7	4	15.4	1	2.9	6	14.3	9	18.0	4	12.1
Christian	5	14.3					3	7.1	1	2.0	2	6.1
Others	3	8.6	2	3.8	2	5.8	1	2.4	2	4.0	3	9.1
Marriage												
Unmarried	35	100	24	92.3	34	100	41	97.6	50	100	29	87.9
Married		85.7	2	7.7			1	2.4			3	9.1
Divorce											1	3.0
Employment												
None	30	14.3	20	76.9	31	91.2	32	76.2	38	76.0	20	60.6
Part‐time	5		5	19.2	3	8.9	9	21.4	12	24.0	8	24.2
Full‐time			1	3.8			1	2.4			5	15.2
Prior employment												
None			7	26.9			19	46.3			7	21.2
Part‐time			5	19.2			13	31.7			7	21.2
Full‐time			14	53.8			9	22.0			19	57.6
Prior BS programme												
Social			10	38.5			22	52.4			15	46.9
Science			1	3.8			3	7.1			4	12.5
Medicine, agriculture			15	57.7			17	40.5			13	40.6

Note.

A, B and C indicating the three schools where we collected the data.

Abbreviations: ABSN, Accelerated Second‐Degree in Nursing Programmes; BSN, Baccalaureate degree.

### 4.2 NPVS‐R

The results of the total score and each subscale of the NPVS‐R for the BSN and the ABSN in each semester were shown in Table 2. For the BSN group, the mean of total scale of the NPVS‐R in the first semester was 110.64 ± 11.91 whereas the mean of total scale of the NPVS‐R in the second semester was 108.48 ± 16.38. The range of mean in each subscale of NPVS‐R during research period was 3.86–4.48.

**TABLE 2:** Self‐comparison between the first and the second semesters for each programme

	BSN						ABSN					
	First semester		Second semester		Paired *t*	*p*	First semester		Second semester		Paired *t*	*p*
	Mean	SD	Mean	SD			Mean	SD	Mean	SD		
Whole subjects												
Caring	4.38	0.47	4.30	0.53	1.57	0.119	4.27	0.50	4.21	0.51	0.47	0.638
Professionalism	4.12	0.64	3.94	0.78	2.64	0.010	4.18	0.71	3.91	0.70	3.19	0.002
Activism	3.86	0.71	3.87	0.75	0.69	0.495	3.98	0.74	3.83	0.71	1.31	0.195
Trust	4.48	0.47	4.43	1.31	1.45	0.149	4.47	0.52	4.27	0.63	3.26	0.002
Justice	4.34	0.67	4.17	0.76	2.33	0.022	4.27	0.65	4.11	0.69	1.56	0.122
Total scale	110.64	11.91	108.48	16.38	*2.05*	0.*043*	110.20	12.92	106.38	13.62	*2.29*	0.*024*
School A												
Caring	38.60	5.16	34.08	5.36	3.47	<0.001	37.27	4.90	37.27	4.90	−0.05	0.96
Professionalism	15.86	2.51	13.69	3.21	2.59	0.02	15.73	2.32	15.73	2.32	0.52	0.61
Activism	19.09	3.64	16.69	3.72	2.59	0.02	18.15	3.46	18.15	3.46	1.25	0.22
Trust	22.20	2.54	18.73	3.96	3.06	0.01	21.62	2.70	21.62	2.70	2.83	0.01
Justice	13.29	2.27	11.12	2.88	2.92	0.01	12.23	2.01	12.23	2.01	0.14	0.89
Total scale	109.03	13.14	94.31	16.61	3.56	<0.001	103.48	11.97	103.48	11.97	1.17	0.25
School B												
Caring	39.90	3.69	39.21	3.68	−1.97	0.06	0.04	0.97	37.84	3.90	0.04	0.97
Professionalism	17.04	2.76	15.81	2.99	−0.69	0.49	1.64	0.11	15.16	3.05	1.64	0.11
Activism	19.76	3.47	19.63	3.52	−1.68	0.10	0.87	0.39	19.50	3.38	0.87	0.39
Trust	22.42	2.48	21.54	2.52	−1.73	0.09	1.52	0.14	21.13	3.10	1.52	0.14
Justice	13.32	1.74	12.46	2.09	−2.53	0.02	0.57	0.58	12.13	2.09	0.57	0.58
Total scale	112.44	11.65	108.65	12.19	−2.49	0.02	1.06	0.29	105.75	13.88	1.06	0.29
School C												
Caring	39.53	4.03	41.46	3.11	1.04	0.30	0.74	0.46	38.64	4.99	0.74	0.46
Professionalism	16.35	2.21	17.08	2.47	2.23	0.03	3.25	<0.001	16.18	2.88	3.25	<0.001
Activism	18.91	3.69	20.68	3.24	−0.08	0.93	0.35	0.73	19.94	3.44	0.35	0.73
Trust	22.53	1.96	25.35	9.68	2.51	0.02	1.84	0.07	21.67	3.59	1.84	0.07
Justice	12.32	1.95	13.46	1.43	2.63	0.01	1.84	0.08	12.76	1.94	1.84	0.08
Total scale	109.65	10.91	118.03	14.21	1.85	0.07	1.85	0.07	109.18	14.38	1.85	0.07

Note.

A, B and C indicated the three schools where we collected the data.

### 4.3 Comparison of the professional value between the ABSN and the BSN programmes

Four types of comparisons were performed, which were comparison of all subjects between the ABSN and the BSN programmes at the same semester; self‐comparison between the first and the second semesters for each programme in three schools; comparison of each programme of the ABSN and the BSN among three schools in each semester; and comparison between the BSN and the ABSN at the same semester in the same school respectively.

#### 4.3.1 Comparison of all subjects between the ABSN and the BSN programmes at the same semester

No significant difference existed in the comparison of the total scale and each subscale between the BSN and the ABSN (*p *> .05) in the first and the second semesters respectively. It showed that similar responses towards the total scale and each subscale were reported in the first semester in the subjects of the BSN and the ABSN. Meanwhile, similar relationship was found in the second semester.

For the subjects in the BSN programme, the results of paired *t* test showed that the total scale, and professionalism and justice subscales had reached the statistical significance of 0.05. It indicated a significant higher score in the total scale, professionalism and justice subscales in the first semester for the subjects in the BSN group. For the ABSN group, the results of paired *t* test showed a significant higher score in the total scale, professionalism and trust subscales in the first semester for subjects in the ABSN group (Table 2).

#### 4.3.2 Self‐comparison between the first and the second semesters for each programme in three schools

For BSN programme, self‐comparison between the first and the second semesters for each school showed a significantly different change in the total scale and subscales (*p* < .05; Table 2). Justice subscale was the only one that showed a change across these comparisons. However, a decreased score was observed in the school A and B, and an increased score existed in the school C.

For the subjects in the ABSN programme, the significantly difference only existed in one subscale of the NPVS‐R in the school A and C. In addition, a significantly higher score was reported in the first semester than that in the second semester for trust and professionalism subscales respectively (*p *< .05).

#### 4.3.3 Comparison of the ABSN programme and the BSN programme among three schools in each semester

Comparison of the BSN programme among three schools showed that no significant differences of the total scale and all subscales of the NPVS‐R existed at the first and the second semesters respectively (*p *> .05; Table 3). The comparison of the ABSN programme showed that, at the first semester, all subscales and the total scale of the NPVS‐R reached the statistical significance of 0.05. It indicated that the subjects in the school A reported a lower score than the school B and C in general. In the second semester, only the subjects in the school A reported significantly a lower activism than the subjects in the school B (*p *= .04; Table 4).

**TABLE 3:** Comparison of the BSN and the ABSN programmes, respectively, among three schools in each semester

	BSN				ABSN					
	First semester		Second semester		First semester			Second semester		
	*F*	*p*	*F*	*p*	*F*	*p*	LSD	*F*	*p*	LSD
Caring	0.97	0.38	1.16	0.32	27.06	<0.001	A < B,C	1.03	0.36	
Professionalism	2.31	0.10	0.75	0.48	10.62	<0.001	A < B,C	1.09	0.34	
Activism	0.67	0.51	1.47	0.23	10.50	<0.001	A < B,C	3.27	0.04	A < B
Trust	0.18	0.84	0.48	0.62	9.41	<0.001	A < B; C < B	0.26	0.77	
Justice	3.03	0.05	0.37	0.69	9.29	<0.001	A < B,C; C < B	1.04	0.36	
Total scale	1.01	0.37	0.85	0.43	22.11	<0.001	A < B,C	1.31	0.28	

Note.

A, B and C indicated the three schools where we collected the data.

**TABLE 4:** Comparison between the BSN and the ABSN programmes at the same semester in the same school

	A				B				C			
	First semester		Second semester		First semester		Second semester		First semester		Second semester	
	*t*	*p*	*t*	*p*	*t*	*p*	*t*	*p*	*t*	*p*	*t*	*p*
Caring	−1.05	0.30	1.94	0.059	0.59	0.56	−2.87	0.01	−1.40	0.16	−1.55	0.13
Professionalism	0.44	0.66	2.42	0.019	−1.05	0.30	−1.41	0.16	−0.31	0.76	−1.05	0.30
Activism	−0.13	0.90	0.96	0.341	−2.01	0.05	−0.92	0.36	0.03	0.97	−0.27	0.79
Trust	0.78	0.44	2.66	0.010	0.23	0.82	−2.06	0.04	−0.65	0.51	−0.65	0.52
Justice	−1.41	0.16	1.34	0.186	−1.36	0.18	−1.74	0.09	−1.04	0.30	−0.81	0.42
Total scale	−0.42	0.67	2.25	0.029	−0.80	0.43	−2.59	0.01	−0.82	0.41	−1.04	0.30

Note.

A, B and C indicating the three schools where we collected the data.

#### 4.3.4 Comparison between the BSN programme and the ABSN programme at the same semester in the same school

Results of *t* tests showed that the only significant differences of the NPVS‐R were existed in the second semester in the School A (*p *< .05; Table 4) and the school B (*p *< .05; Table 4). In the school A, the subjects in the ABSN programme reported higher scores in the total scale, and professionalism and trust subscales of the NPVS‐R than these in the BSN programme. In the school B, the subjects in the ABSN programme reported lower scores in the total scale, and caring and trust subscales of the NPVS‐R than the subjects in the BSN programme.

## 5 DISCUSSION

### 5.1 Characteristics of the samples

The only significant difference of demographic characteristics was that the subjects in the ‘ABSN’ programme were significantly older than the subjects in the ‘BSN’ programme. This fits the fact that the subjects in the ABSN programme need to obtain their first bachelor degree; then, they are qualified to enter this new ASBSN programme.

Some students in the ABSN worked as part‐time, even full‐time, while they were studying this new nursing programme, it might indicate the independent needs and financial burden of these students as found in other countries (Kowitlawakul et al., 2013; Meyer et al., 2006). From the disciplines of their previous BS programme, most of them were in social sciences which was similar to the subjects in USA (Meyer et al., 2006). Compared with the nursing profession, the occupations related to these social sciences have been recognized as a career with limited vacancy and lower salary in general. These might be the reasons for them to enter this new ABSN programme, even though additional 3‐year studies are required.

### 5.2 Results of NPVS‐R

#### 5.2.1 Comparison of all subjects between the ABSN programme and the BSN programmes at the same semester

Various types of results were reported for the NPVS‐R in literature. For example, some studies reported the total score only (Alfred et al., 2013; Iacobucci et al., 2013; Lin et al., 2010, 2016; Poorchangizi et al., 2019a), the subscales only (Bijani et al., 2019; Caldwell & Katie, 2016), did not follow the original format to calculate the total score (Posluszny & Hawley, 2017), or used different classification of subscales from the original suggestions (Bang et al., 2011; Culha & Acaroglu, 2019; Lin et al., 2010, 2016).

In this study, the range of mean score of the NPVS was 106.38 ~ 110.64. Similar mean score was reported in the nursing students from America as 109.2 ± 12.3 (Alkaya et al., 2018) and 106.16 ± 12.93 (Alfred et al., 2013) and from Turkey as 106.4 ± 13.6 (Geçkil et al., 2012), whereas a lower mean score reported by nursing students in China as 100.47 ± 16.6 (Lin et al., 2016), in Taiwan as 104.27 ± 16.81 (Alfred et al., 2013) and 99.10 ± 15.6 (Lin et al., 2016), in Turkey as 103.25 ± 16.96 (Ayla et al., 2018) and 101.6 ± 17.0 (Alkaya et al., 2018) and in America as 101.43 ± 12.78 (Iacobucci et al., 2013). Although culture had been identified as a factor associated with the professional value (Alfred et al., 2013; Alkaya et al., 2018), different results existed even though the data were collected from the same country. For example, our data (106.38 ~ 112.44) were greater than 99.10 ± 15.6 (Lin et al., 2016) and 104.27 ± 16.81 (Alfred et al., 2013) that were collected in Taiwan. It might be associated with the promotion of social status and salary of nurses during last decade in Taiwan. Further exploration is needed.

Meanwhile, the subjects in both the BSN programme and the ABSN programmes reported a similar level of the professional value when they began their learning of professional nursing. In the western literature, students in the ABSN programme were recognized as highly independent and motivated (Penprase & Koczara, 2009). They also reported a higher level of the professional value than students in the BSN programme (Fernandez‐Feito et al., 2019). In our samples, these students in the ABSN programme competed with many candidates and earned the opportunity to enter this new programmes. Similar situations existed in students of the BSN programme who defeated many challengers in the Nationwide Entrance Examination. Strong motivation and willing to be professional nurses might explain the similar levels of the professional value when they started their learning in nursing profession.

The results of present study also found that the total score and two subscales of the NPVS in the first semester were significantly higher than those in the second semester for subjects both in the BSN programme and the PBSN programmes. For each school, similar nursing courses and learning experiences were provided during the study period. These subjects received their fundamental nursing and the first clinical practicum during the second semester. To learn and to see the reality of nursing theory and practice might break their imagination and dream of ‘Angel in white’. These might reduce their professional value in return. However, the reduced dimensions of the NPVS in the BSN programme and the PBSN programme need further exploration.

##### Self‐comparison between the first and the second semesters for each programme in three schools

The self‐comparisons in the BSN programme, except in the school C, reported higher values in certain subscales and the total scale in the NPVS‐R in the 1st semester even though our follow‐up was one semester only. Similar trend was found in one subscale of the NPVS‐R in the ABSN programme in school A and C. Contradictory results were found in literatures that the professional values increased with time (Fisher, 2014; Kantek et al., 2017; Leners et al., 2006; Lin et al., 2010). As mentioned previously, learning professional nursing might increase their stress and understanding of reality in nursing care. Therefore, they reported a lower value and passion to be a nurse. Will faculties and learning environment be the reasons? Further exploration is needed.

The subjects in the school B, both the BSN pram and the ABSN programme, reported a similar level of the NPVS‐R. Literature did find that there was no difference during school year (Caldwell & Katie, 2016; Poorchangizi et al., 2019a; Posluszny & Hawley, 2017). The school B is a private school, and the same group of faculties taught these two programmes. Thus, faculties might be a reason related to the perceived levels of changes in the professional value.

##### Comparison of each programme among three schools in each semester

Present study showed that no significant differences existed in the NPVS‐R for the BSN subjects in three schools at the first and the second semesters respectively. It might be associated with the facts that the Ministry of Education in Taiwan regulates the curriculum of each nursing school in order to fulfil the requirements of the Nursing License Examination. Subjects in the BSN programme received similar nursing courses and learning experiences after they passed the National Entrance Examination and entered into these nursing programs. In addition, these three schools are experienced in teaching this program. Similar levels of the professional value are acceptable.

For the ABSN programme, the subjects in the school A reported a lower score in the NPVS‐R than those in the school B and C in general. However, in the second semester, only the subjects in school A reported significantly lower activism than the subjects in school B. We have mentioned that the school A just started this new programme. Each school recruited their students in the ABSN programme independently. Different sources of students, previous learning and working experiences of students, and their lived experiences might have impact on their learning experiences and perception of nursing. Thus, levels of the professional value might be differently reported even though they just started their leaning in nursing profession. After one semester's learning, the students in school A catch up with the students in the other two programs, and activism was the only subscale reported lower than the students in school B. The improvements might be associated with the teaching and coaching efforts of faculties in school A that has been recognized as top one in Taiwan. It also indicated that progression of the professional values is possible through education.

##### Comparison between the BSN programme and the ABSN programme at the same semester in the same school

Our study showed that, in school A, subjects in the ABSN programme reported a higher level of professional value then subjects in the BSN programme. It was supported by literature (Feller et al., 2019). Contradictory result was found in school B. However, for school C, a similar level of the professional value was reported and fitted the literature (Astorino, 2006).

Similar curriculum and environments existed in these three schools, except the school A is a public school and just starts their first year of teaching for this new ABSN programme and the other two schools are private schools with 2 years’ experiences of teaching this new programme. Will the policy of school, environments, characters and experiences of facilities make the differences? Further exploration is needed.

### 5.3 Limitations and suggestions for further study

The limitation of the present study was that these results cannot be generalized into the whole nursing programmes during the whole academic years because it only collected one class of subjects from 3/5 nursing schools and during one academic year. Larger sample sizes and longer duration of follow‐up are strongly suggested for further study. Thus, we can get a whole picture of the development of the professional value for students both in the BSN programme and the ABSN programme that have never been explored yet. Improving the professional value was possible from our findings with 1‐year follow‐up. Redesign teaching strategies to fit the characters of the students in the ABSN programme was strongly suggested. These might promote the development of the professional value and help the students in this new programme reach the ultimate goals of learning in nursing profession.

## 6 CONCLUSIONS

New type of the nursing educational programme was implemented in Taiwan recently. Students in the BSN and the ABSN programmes were purposely sampling and recruited during one academic year. The returned questionnaires showed the various patterns of changes in the professional value in each comparison with types of programmes and schools of nursing. Also, the increased professional value in the school A showed the possibility of improvement during the first learning of professional nursing. As educators, we must redesign our teaching strategies according to the different conditions of the students in each programme.

## CONFLICT OF INTEREST

The authors declare that they have no competing interests.

## AUTHOR CONTRIBUTIONS

Hui‐Ping Lin; Hsueh‐Erh Liu: Conceptualization and design of the study, and manuscript writing and revision. Tsai‐Hsiu Chang, Hsiu‐Fang Lee, C.W Chang and Hui‐Chu Chiang: Data collection. Hsiu‐Fang Lee, Yu‐Hsia Tsai, C.W Chang and Hui‐Chu Chiang: Data analysis. Tsai‐Hsiu Chang and Hsueh‐Erh Liu: Supervision of the whole study process. All the authors have read and approved the final version of the manuscript.

## Data Availability

The data that support the findings of this study are available from the corresponding author upon reasonable request.
